# Clinical outcomes of synthetic absorbable mesh use in breast surgery: First case series in reconstruction and aesthetic mastopexy

**DOI:** 10.1016/j.jpra.2026.03.030

**Published:** 2026-03-27

**Authors:** Leonardo Adolfo Spuras Stella, Bruna Lee Damasceno, Giovanna Valente Costa, Nicole Martins Stella, Vanessa Borges Valente Stella, Rosa Andréa Nogueira Laiso, Rose Eli Grassi Rici, Gustavo Henrique Doná Rodrigues Almeida, Durvanei Augusto Maria

**Affiliations:** a Graduate Program in Medical Sciences, School of Medicine, University of São Paulo (USP), Av. Dr. Arnaldo, 455, São Paulo, SP, Brazil; b Private Practice, Clínica Stella, Americana, SP, Brazil; c Pontifical Catholic University of Campinas (PUC-Campinas), Rod. Dom Pedro I, km 136, Campinas, SP, Brazil; d Graduate Program in Anatomy of Domestic and Wild Animals, School of Veterinary Medicine and Animal Science, University of São Paulo (USP), Av. Prof. Dr. Orlando Marques de Paiva, 87, São Paulo, SP, Brazil; e Laboratory of Development and Innovation, Butantan Institute, Av. Vital Brasil, 1500, São Paulo, SP, Brazil

**Keywords:** Breast reconstruction, Biomaterial, Synthetic absorbable mesh, Plastic surgery

## Abstract

Breast cancer remains one of the leading causes of female mortality worldwide, and mastectomy is often required as a therapeutic or prophylactic measure. Breast reconstruction plays a key role in restoring body symmetry and quality of life. Synthetic meshes have recently gained attention as adjuncts intended to provide additional soft-tissue support and assist in implant stabilization. Among these, the absorbable GORE^Ⓡ^ BIO-A^Ⓡ^ FS0915 has shown favorable results in soft tissue reconstructions, but its use in breast surgery has not been reported. This retrospective study included women who underwent breast reconstruction or mastopexy with implants and absorbable matrix between October 2022 and October 2024 at three hospitals in Americana, Brazil. Data were retrospectively collected and included demographic, clinical, surgical, and adjuvant treatment variables. Complications were classified as minor (not requiring reintervention) or major (requiring surgical management). Descriptive statistics were used to summarize the data. Thirty-eight patients (66 breasts) were included. Patient-level characteristics were analyzed per patient (*n* = 38), while surgical outcomes and complications were analyzed per breast (*n* = 66). The mean age was 47 years, and complications (per breast) were observed predominantly in older patients, those with multiple prior surgeries, and those exposed to radiotherapy or chemotherapy. Breast cancer was the main indication (79%), and no complications were observed in aesthetic-only cases. The overall complication rate was 19.7%, with 7.6% major (contracture, infection) and 12.1% minor (seroma, edema, rotation, dehiscence). In summary, the use of synthetic mesh in breast reconstruction and mastopexy was technically feasible and showed an acceptable preliminary safety profile within the context of this retrospective case series.

## Introduction

Breast cancer is one of the leading causes of female mortality worldwide and shows an increasing incidence. According to global epidemiological statistics from the World Health Organization (WHO), it is estimated that between 2024 and 2025 > 2.3 million women will be diagnosed with the disease.^1^^,^^2^ Among these patients, about 40% will undergo mastectomy as a prophylactic or therapeutic measure to prevent cancer progression.^3^^,^^4^ Although surgical intervention remains a cornerstone of breast cancer treatment, its indication is currently guided by personalized oncologic and reconstructive considerations, reflecting the high survival rates of modern therapy. Even in developed countries, where early diagnosis is more frequent, the disease still accounts for more than 1 million deaths annually, reinforcing the urgent need for continuous improvement of treatment and prevention strategies.^5^^,^^6^

In order to restore the structural and aesthetic integrity of the removed breasts, breast reconstruction is performed, considered an essential step in the rehabilitation process of patients after mastectomy.^7^ This procedure is not limited to the replacement of breast volume, but also involves the recovery of body symmetry and female self-esteem, directly impacting quality of life.^8^ Reconstruction can be carried out through different approaches, such as the use of silicone implants, tissue expanders, or autologous tissues, depending on the clinical conditions of the patient, the stage of the disease, and personal preferences.^9^ Among the available techniques, the use of synthetic and semi-synthetic matrices has gained prominence as a promising alternative to optimize the aesthetic and functional outcomes of breast reconstruction.^10^^,^^11^ These matrices act as temporary scaffolds, providing greater mechanical stability to the adjacent tissue and promoting integration of the implant with the surrounding tissues.^12^ Such biomaterials are generally biocompatible and biodegradable, not inducing exacerbated inflammatory responses or immunological rejection, which allows the formation of new tissue in the medium and long term.^12^^,^^13^ This property is particularly relevant in patients with limited tissue coverage or those requiring additional support due to extensive resections.^14^ The application of these matrices has been explored as a strategy to provide structural support in implant-based reconstruction, although reported outcomes vary across studies.^15^ Another important benefit is the potential reduction in surgical time when compared to techniques that employ autologous tissues. Nevertheless, possible disadvantages must be considered, such as the risk of infection, seroma formation, and implant exposure, which underscores the importance of careful patient selection to ensure the success of the procedure.^16^

The choice between synthetic or biological matrices should take into account a series of clinical and individual factors that directly influence the success of breast reconstruction, including the extent of the surgical resection, the quality and thickness of the remaining tissues, the presence of systemic comorbidities, as well as a previous history of radiotherapy or chemotherapy, which may compromise healing and implant integration.^17^ Aspects related to patient profile, such as age, aesthetic expectations, tolerance to additional procedures, overall surgical risk, and availability of local tissue support, should also be considered.^18^ From a surgical perspective, additional factors include the desired operative time, the experience of the surgical team with each technique, the likelihood of complications at donor sites, and the overall costs involved in the procedure.^19^

Synthetic matrices are produced under controlled industrial conditions, with consistent mechanical and structural properties, which reduces the biological variability inherent to matrices derived from human or animal tissues.^20^^,^^21^ In addition, there is no need for donor harvesting or ethical constraints regarding their use.^21^ They can also be engineered with specific properties, such as controlled porosity, adjusted mechanical strength, programmed degradation rate, and even incorporation of bioactive molecules, which provides greater adaptability to the type of surgical procedure and to patient-specific characteristics.^22^

Therefore, considering the technical, operational, and biological advantages of synthetic matrices, the present study proposes a new surgical approach for breast reconstruction and aesthetic mastopexy using the absorbable GORE^Ⓡ^ BIO-A^Ⓡ^ FS0915 matrix. This biomaterial is already well established in reconstructive procedures of soft tissues, such as abdominal wall repair and the treatment of fistulas, but to date no clinical reports have documented its application in breast reconstruction surgery. Thus, this study aimed to demonstrate the feasibility of its use in this context, to analyze its correlation with patient profiles, and to investigate potential complications associated with the technique.

## Methodology

### Study design

**Figure 1:**
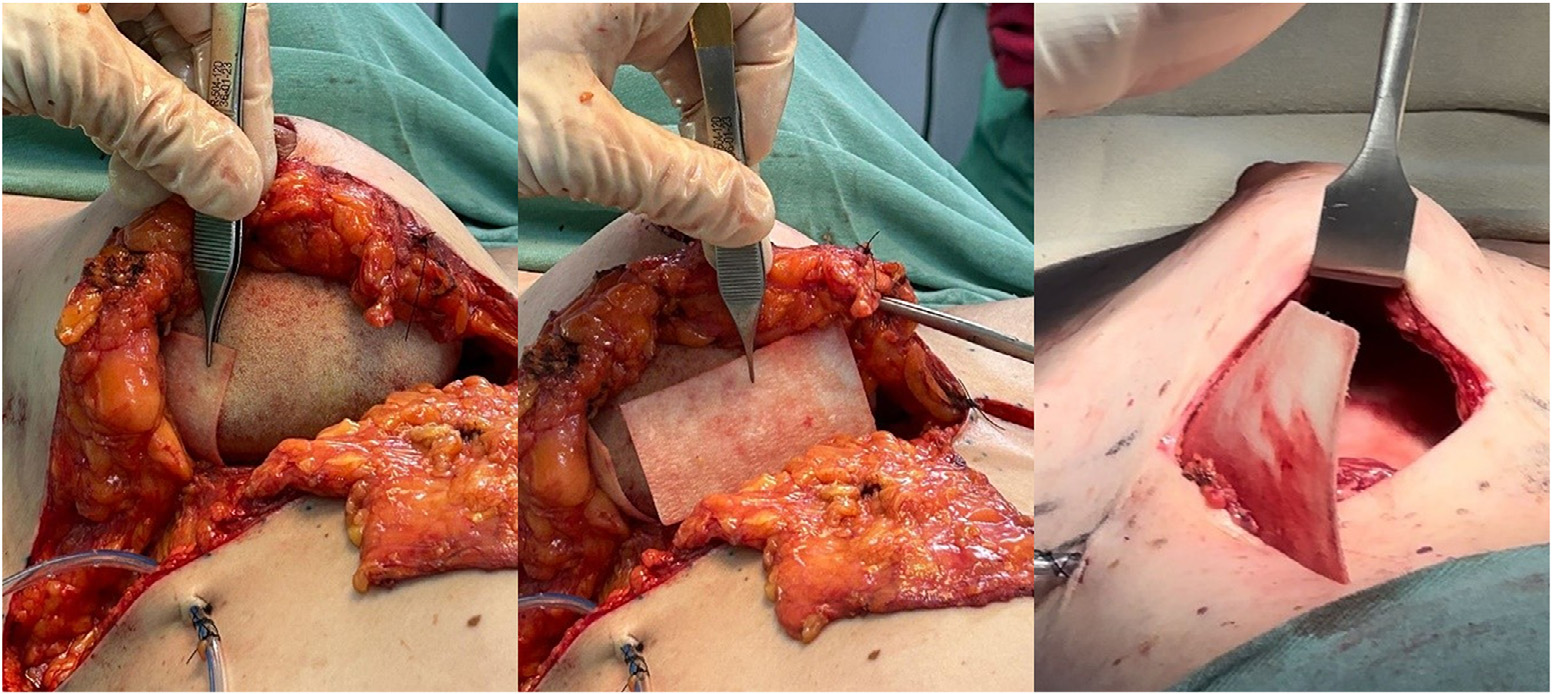
Placement of the synthetic mesh was performed in different surgical planes, always positioned in close contact with the implant to provide reinforcement and enhance reconstruction stability. In the subglandular plane covering the lateral pole of the breast, the mesh acted as additional support by reducing tension on the glandular tissue and improving the lateral contour (left panel). When placed in the subglandular plane covering the inferior pole, it functioned as a hammock for the implant, preventing inferior migration (“bottoming out”) and preserving the integrity of the inframammary fold (middle panel). Finally, in the submuscular plane covering the lateral pole, the mesh reinforced the muscle–implant interface, contributing to implant stability and minimizing the risk of lateral displacement (right panel).

The present study is a retrospective analysis of a consecutive series of all patients, with no case selection, who underwent breast reconstruction or aesthetic mastopexy with implants combined with the absorbable synthetic matrix GORE^Ⓡ^BIO-A^Ⓡ^FS0915, a bioabsorbable synthetic scaffold composed of a copolymer of polyglycolic acid (PGA) and trimethylene carbonate (TMC), designed to provide temporary soft-tissue reinforcement during the early phases of healing, with progressive degradation over approximately 6 to 7 months^23^ (Figure 1), performed between October 2022 and October 2024 in three hospitals located in Americana, São Paulo, Brazil. Data were obtained through a structured review of medical records, encompassing clinical, surgical, and postoperative follow-up information of the included patients. The Institutional Ethics Committee was informed about the retrospective nature of the study and confirmed that formal ethical approval was not required according to local regulations. Written informed consent for the publication of identifiable photographs was obtained from all patients whose images appear in this article. The manuscript was prepared in accordance with the Strengthening the Reporting of Observational Studies in Epidemiology (STROBE) guidelines (see Supplemental Appendix).

### Surgical procedures

All procedures were performed under general anesthesia, with a single intravenous dose of cefazolin (2 g) administered at induction for antibiotic prophylaxis, along with 1 g of intravenous tranexamic acid (ADVANZ Pharma, London, UK). Skin preparation was carried out in two stages using 2% chlorhexidine gluconate (Ecolab, Andover, UK). When inframammary scars were present, they were excised. Previously inserted implants were removed, and the manufacturer, size, and model were recorded. Clinical photographs were obtained, with patient consent, for medical documentation.

Capsular management varied according to the anatomical plane of the previous implant. In cases of subglandular placement, a complete capsulectomy was performed and the capsule was sent for histopathological analysis, including specific evaluation for BIA-ALCL. For implants located in the submuscular or dual plane, efforts were made to remove the entire capsule without excessive trauma to the ribs, intercostal muscles, or lower portion of the pectoralis major. When the capsule was densely adherent or unsafe to remove in its entirety, representative samples were collected for histology.

When a new subglandular pocket was created to replace a previous submuscular implant, the pectoralis major muscle was reattached to its origin using a continuous locking 3/0 polydioxanone suture (Ethicon PDS II; Ethicon Inc., Somerville, NJ, USA). Regardless of the plane, meticulous hemostasis of the pocket was always performed prior to placement of the absorbable synthetic matrix (GORE^Ⓡ^ BIO-A^Ⓡ^ FS0915). The definitive implant was then introduced using an insertion sleeve to minimize trauma or displacement of the matrix, and reinforcement of the inframammary fold as well as the lateral component of the mammary ligament was performed with absorbable sutures to optimize inferior and lateral pole support. Wound closure was carried out in multiple layers with polyglycolic acid (Vicryl^Ⓡ^) and poliglecaprone 25 (Monocryl^Ⓡ^) sutures, followed by application of skin adhesive systems (Dermabond^Ⓡ^ or Dermabond Prineo^Ⓡ^, Ethicon Inc., Somerville, NJ, USA). Drain placement was systematic in reconstruction procedures and, in aesthetic cases, was left to intraoperative judgment. In selected patients, autologous fat grafting was performed in the subdermal plane either focally or throughout the breast to enhance coverage and contour.

### Breast reconstruction

Breast reconstruction was performed in two stages. In the first stage, following mastectomy, a tissue expander was positioned beneath the pectoralis major. After progressive expansion, the second stage consisted of expander removal and replacement by a permanent implant through an inframammary incision of approximately 8 cm. The pectoralis major was released along its inferior border, the expander and valve were removed, and superior and medial capsulotomies were carried out to accommodate the new implant. The prosthesis was placed in a dual plane, with the upper and lateral poles submuscular and the lower pole subglandular. The absorbable synthetic matrix, trimmed according to the dimensions of the lower pole, was positioned to provide reinforcement of the lateral breast and additional coverage of the inferior aspect of the implant. Layered closure was then performed.

### Aesthetic mastopexy or contralateral reconstruction

Aesthetic mastopexy with implant placement was performed either in the subglandular or dual-plane position, depending on anatomical and clinical indications. Preoperative markings included the nipple–areola complex (Pitanguy’s point A) and the amount of redundant skin to be excised. In both approaches, bilateral Schwartzmann maneuver was performed, combined with the resection of inferior glandular tissue and creation of a dermoglandular flap approximately 0.5 cm thick, with an 8 cm base and variable height (4–10 cm). Hemostasis was reviewed carefully, and inframammary and lateral support were reinforced with absorbable sutures (Vicryl^Ⓡ^ 1.0 and 3.0). In the subglandular technique, polyurethane implants were positioned directly in the pocket, with the synthetic matrix reinforcing the lateral and inferior poles. Although polyurethane-coated implants typically show good tissue integration, the mesh was used in these cases as an adjunctive reinforcement strategy, according to surgeon preference and intraoperative assessment. In the dual-plane technique, a 5 cm incision was made at the inferior border of the pectoralis major, which was partially released to allow placement of a microtextured implant, maintaining the inferior portion subglandular. Additional glandular resection was performed to reduce volume and balance symmetry. In both approaches, the nipple–areola complex was transposed on a superior or superomedial pedicle and fixed at the pre-marked point A. The absorbable synthetic matrix was positioned to reinforce lateral and inferior coverage, ensuring implant stability. Closure was completed in layers with Vicryl^Ⓡ^ 4.0 and Monocryl^Ⓡ^ 4.0.

### Postoperative care

In the immediate postoperative period, all patients were fitted with a surgical compression bra, supplemented with a thoracic strap or binder when indicated by the type of procedure. The duration of external support varied between 1 and 5 weeks. Post-operative antibiotic prophylaxis consisted of oral cephalexin 500 mg every 6 h for 7 to 10 days, and analgesics were prescribed according to individual requirements. The duration of postoperative antibiotic prophylaxis reflected institutional practice patterns and surgeon preference during the study period, particularly considering the use of implantable biomaterial. This approach was adopted as a precautionary strategy and should not be interpreted as a universal recommendation. Postoperative follow-up included evaluations at 24 h, 1 week, and 3 weeks, during which motor physiotherapy and lymphatic drainage were performed twice weekly for three consecutive weeks. Long-term follow-up was conducted at 6 months and 1 year, after which patients were discharged, with open access to further consultations if necessary. The median follow-up period was 18 months (range 8–32 months) for reconstructive cases and 14 months (range 6–28 months) for aesthetic cases.

### Data collection

Data were retrospectively collected from medical records in January 2025. Patient-level variables included age, weight, height, and body mass index (BMI), as well as lifestyle factors such as smoking status. Clinical history comprised the number of prior breast procedures and the indication for the current surgery. Information regarding neoadjuvant or adjuvant treatments, including radiotherapy and chemotherapy, was also recorded. Postoperative complications were predefined and categorized as minor (not requiring surgical intervention) or major (requiring surgical management). Capsular contracture was clinically assessed during follow-up and classified according to the Baker grading system; only grade III or IV cases were considered clinically relevant. Infection was defined by the presence of local inflammatory signs associated with the need for systemic antibiotic therapy and/or surgical intervention. Seroma was defined as a fluid collection requiring aspiration or drainage. Edema was considered a complication when persistent beyond the expected postoperative course. Wound dehiscence was defined as partial separation of the surgical incision requiring clinical management. Implant rotation was diagnosed clinically when malposition resulted in visible asymmetry.

### Data analysis

Data were organized using Microsoft Excel^Ⓡ^ and analyzed descriptively. Categorical variables are presented as absolute numbers and percentages, while continuous variables are expressed as mean ± standard deviation or median with interquartile range (IQR), as appropriate. Due to the limited number of complication events, particularly major complications, no inferential statistical analyses were performed in order to avoid unstable estimates. Analyses were conducted at two levels: per patient (*n* = 38) and per breast (*n* = 66). Demographic and clinical characteristics were analyzed at the patient level, whereas surgical variables and postoperative outcomes were analyzed at the breast level, as each breast represents an independent surgical unit. This distinction is explicitly maintained throughout the Results section to ensure clarity in data interpretation.

## Results

Table 1 summarizes the demographic and clinical characteristics of the 38 patients (patient-level analysis). Data are presented according to the presence of complications per patient (*n* = 25 without complications, *n* = 8 with minor complications, and *n* = 5 with major complications). The mean age was 47.0 ± 10.4 years, with patients in the major complications group being older (55.2 ± 2.6 years) compared to those without complications (45.8 ± 10.6 years). All patients were non-smokers. The mean BMI was 26.7 kg/m², and the majority of women were overweight (*n* = 21). Regarding surgical history, most patients had previously undergone at least one breast procedure. Multiple prior surgeries were more frequent among patients with major complications: three had undergone two previous surgeries and two had undergone three. The primary indication for surgery was breast cancer and reconstruction (79%), and all major complications (per breast) occurred in this group. In contrast, aesthetic and reconstructive indications accounted for 21% of cases, with no complications reported in this subgroup. Preoperative radiotherapy was performed in 24 patients (63.2%) and was more prevalent among those with complications (4 out of 5). Adjuvant chemotherapy was administered to 25 patients (65.8%) and was present in all patients with complications.

**Table 1:** Demographic and clinical characteristics of patients undergoing breast reconstruction or aesthetic mastopexy with synthetic mesh (*n* = 38).

Variables	All (*n* = 38)	No complications (*n* = 25)	Minor complications (*n* = 8)	Major complications (*n* = 5)
	*n* (%) or mean ± SD	*n* (%) or mean ± SD	*n* (%) or mean ± SD	*n* (%) or mean ± SD
*Age, years*	47.02 ± 10.36	45.12 ± 10.87	46.75 ± 8.94	55.20 ± 2.58
*Non-smoker*	38 (100%)	25 (100%)	8 (100%)	5 (100%)
*Weight (kg)*	71.83 ± 13.69	73.10 ± 14.92	70.00 ± 11.33	67.50 ± 9.14
*Height (m)*	1.64 ± 0.07	1.65 ± 0.07	1.63 ± 0.06	1.58 ± 0.04
*BMI (kg/m²)*	26.65 ± 4.39	26.80 ± 4.82	26.11 ± 3.76	26.80 ± 2.71
Underweight	1 (2.6%)	1 (4.0%)	0 (0.0%)	0 (0.0%)
Normal weight	14 (36.8%)	9 (36.0%)	4 (50.0%)	1 (20.0%)
Overweight	17 (44.7%)	10 (40.0%)	4 (50.0%)	3 (60.0%)
Obesity	4 (10.5%)	3 (12.0%)	1 (12.5%)	0 (0.0%)
*Previous breast surgeries*	1.89 ± 0.76	1.68 ± 0.69	2.00 ± 0.76	2.40 ± 0.55
None	2 (5.3%)	2 (8.0%)	0 (0.0%)	0 (0.0%)
One	7 (18.4%)	6 (24.0%)	1 (12.5%)	0 (0.0%)
Two	22 (57.9%)	14 (56.0%)	5 (62.5%)	3 (60.0%)
Three	7 (18.4%)	3 (12.0%)	2 (25.0%)	2 (40.0%)
*Surgical indication*				
Neoplasia and reconstruction	30 (79.0%)	17 (68.0%)	8 (100%)	5 (100%)
Aesthetic and reconstruction	8 (21.0%)	8 (32.0%)	0 (0.0%)	0 (0.0%)
*Preoperative radiotherapy, yes*	24 (63.2%)	13 (52.0%)	7 (87.5%)	4 (80.0%)
*Adjuvant chemotherapy, yes*	25 (65.8%)	12 (48.0%)	8 (100%)	5 (100%)

Note: BMI: body mass index.

**Figure 2:**
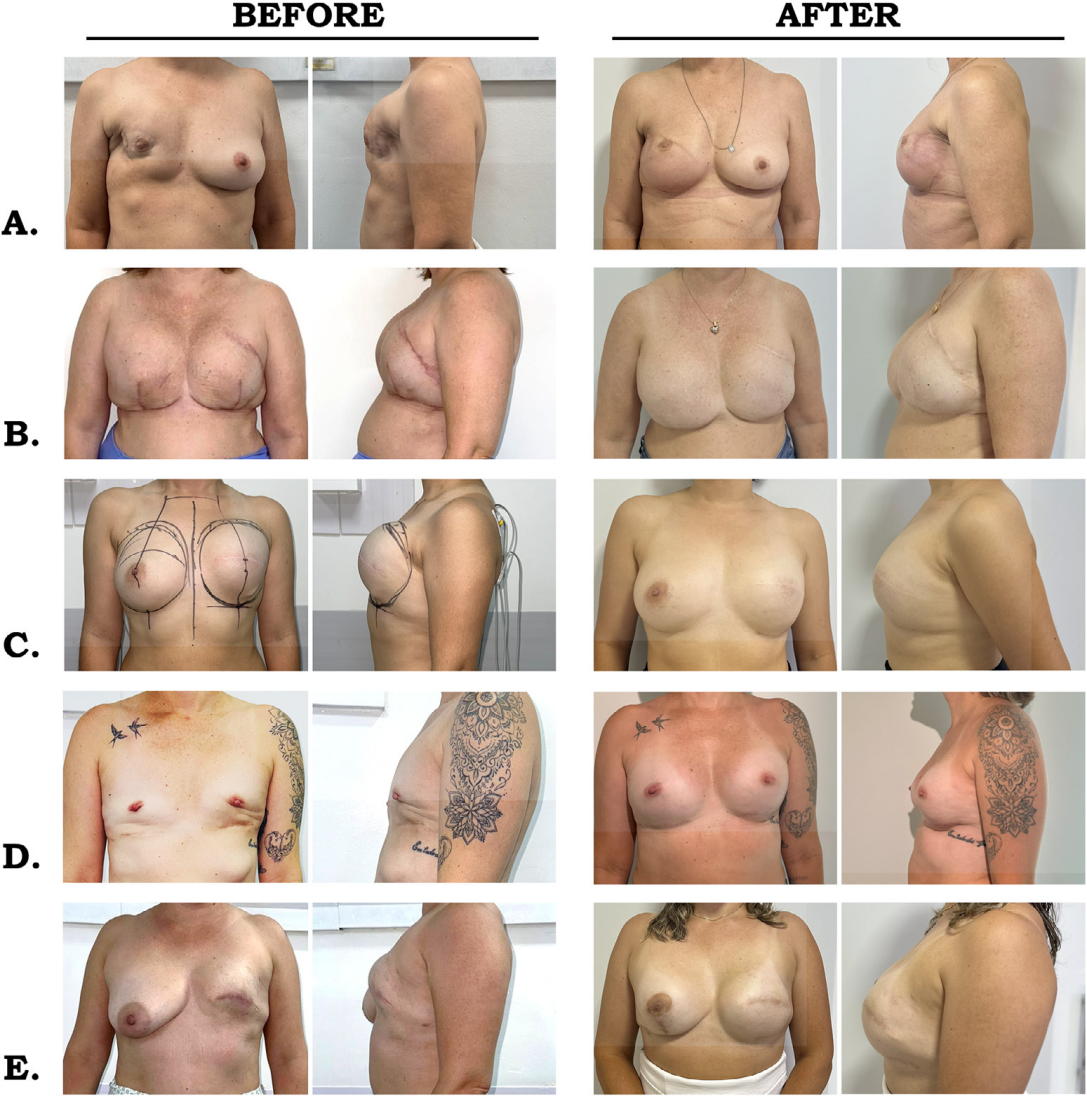
Preoperative and postoperative photographs of patients undergoing breast reconstruction with synthetic mesh. Preoperative images (left column) show sequelae of mastectomy, including volume deficiency, ptosis, contour irregularities, and asymmetry. Postoperative images (right column), obtained 6 to 12 months after surgery, demonstrate increased breast volume, greater projection, improved definition of the lower pole and inframammary fold, and more symmetric breast configuration across cases (A–E).

Figure 2 shows different pre- and postoperative presentations of breast reconstructions performed with the synthetic mesh. In Figure 2A, the preoperative images demonstrate hypomastia on the left side, with slight asymmetry and upper pole deficiency, while the postoperative images reveal restoration of breast volume, improved projection, and a more balanced contour. In Figure 2B, preoperative scars and contour irregularities were associated with volume asymmetry; after reconstruction, breast leveling, smoother contour, and better positioning of the nipple–areola complex were achieved. Figure 2C shows moderate ptosis, low-positioned areolas, and lack of upper pole fullness preoperatively; postoperatively, ptosis correction, upper pole volume restoration, and natural projection are evident, with improved symmetry. In Figure 2D, marked hypomastia and asymmetry were corrected with volume restoration, improved upper pole fullness, and enhanced symmetry. Figure 2E demonstrates breast hypoplasia and mild ptosis, with a poorly defined lower contour; after surgery, volume gain, adequate projection, and improved lower pole definition were observed.

**Figure 3:**
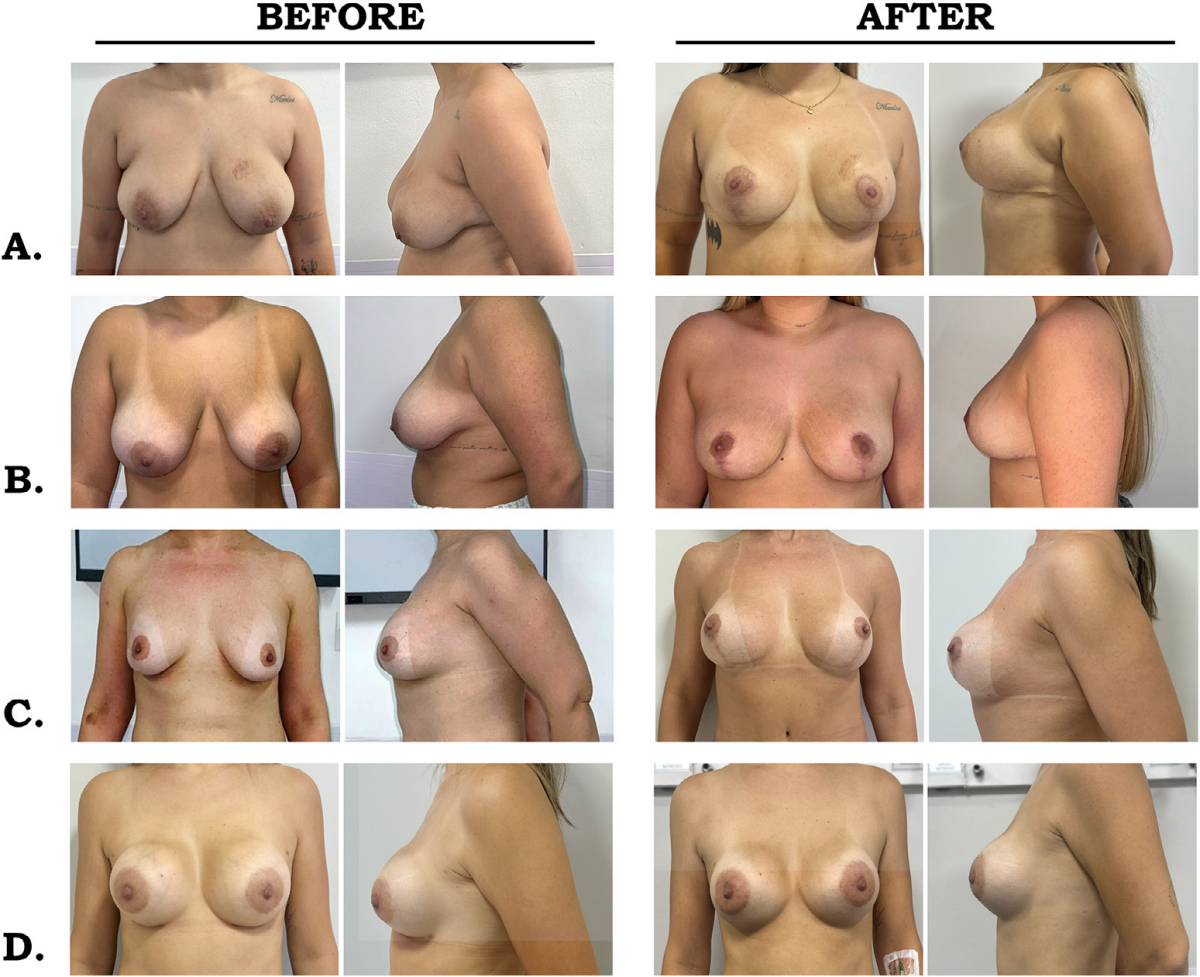
Pre- and postoperative photographs of patients undergoing aesthetic mastopexy with synthetic mesh. In the preoperative images (left column), varying degrees of breast ptosis, volume asymmetry, and poorly defined lower contour are observed. In the postoperative images (right column), obtained approximately 12 months after surgery, improved breast positioning and projection can be seen, with enhanced upper pole fullness, better definition of the lower pole and inframammary fold, and greater symmetry across the different cases (A–D).

Figure 3 illustrates the outcomes of patients who underwent mastopexy procedures with the use of synthetic mesh. In Figure 3A, the preoperative images show marked ptosis, loss of upper pole fullness, and a low inframammary fold; postoperatively, there is breast elevation, restoration of upper pole volume, and improved thoracic contour. In Figure 3B, the breasts were large with grade II ptosis and mild volume asymmetry, which were corrected by repositioning of the nipple–areola complex, redistribution of breast tissue, and achievement of a more harmonious contour. In Figure 3C, preoperative asymmetry and breast flaccidity with deficient upper pole fullness evolved into greater projection, recovery of upper fullness, and enhanced symmetry after surgery. Finally, Figure 3D shows moderate ptosis and flattening of the upper pole preoperatively, corrected postoperatively with nipple–areola elevation, restoration of upper pole volume, and a more natural and proportional breast shape.

**Figure 4:**
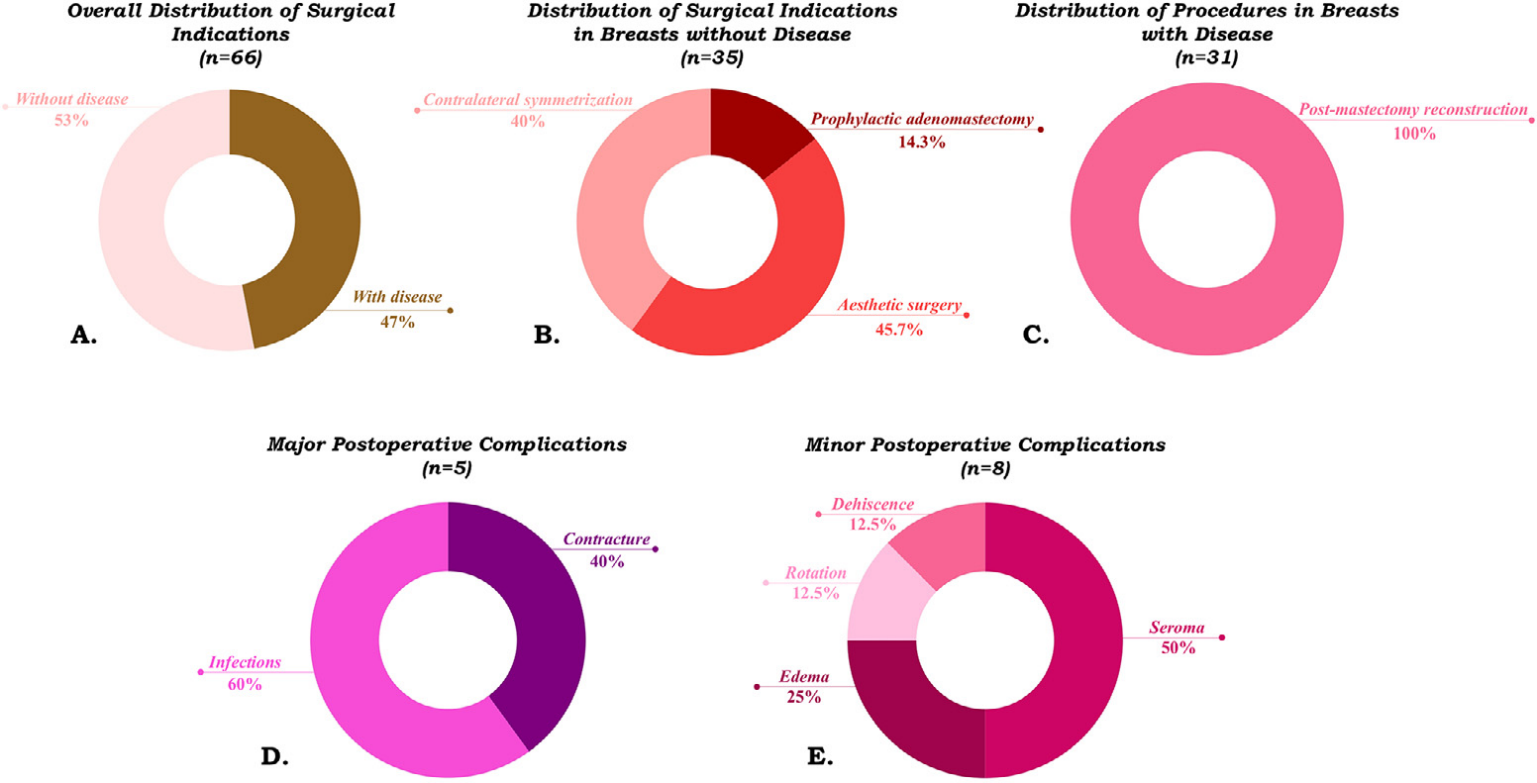
Distribution of surgical indications and postoperative complications in patients undergoing procedures with synthetic mesh. Of the 66 operated breasts, 31 were in patients with disease (47%) and 35 in those without disease (53%) (A). Among the 35 breasts without disease, 5 underwent prophylactic adenomastectomy (14.3%), 16 aesthetic procedures (45.7%), and 14 contralateral symmetrization surgeries (40.0%) (B). In the 31 breasts with disease, all interventions corresponded to post-mastectomy reconstructions (100%) (C). The overall prevalence of complications was 19.7% (13 of 66 cases), with 5 major complications (7.6%) and 8 minor complications (12.1%). Major complications included 2 cases of contracture (40%) and 3 cases of infection (60%) (D). Minor complications included 4 cases of seroma (50%), 2 of edema (25%), 1 of implant rotation (12.5%), and 1 of dehiscence (12.5%) (E).

A total of 66 breasts (breast-level analysis) were operated on using synthetic mesh, of which 31 (47%) were in patients with disease and 35 (53%) in patients without disease (Figure 4A). Among the breasts without disease, 5 (14.3%) underwent prophylactic adenomastectomy, 16 (45.7%) aesthetic procedures, and 14 (40.0%) contralateral symmetrization surgeries (Figure 4B). In the 31 breasts with disease, all interventions consisted of post-mastectomy breast reconstruction, aimed at restoring breast shape and aesthetics following oncologic treatment (Figure 4C). The overall prevalence of postoperative complications per breast was 19.7% (13/66 breasts), of which 5/66 (7.6%) were major complications and 8/66 (12.1%) were minor complications. Among the major complications, two cases of contracture (40%) and three cases of infection (60%) were observed (Figure 4D). Among the minor complications, there were four cases of seroma (50%), two of edema (25%), one of implant rotation (12.5%), and one of dehiscence (12.5%) (Figure 4E).

## Discussion

The use of synthetic or biologic meshes for reconstructive surgery has become increasingly frequent, particularly in breast reconstruction after mastectomy.^24^ These materials provide additional support to the implant, offering greater stability and improved definition of the breast poles, especially the inferior and lateral regions where native tissue is often insufficient.^25^ From a practical standpoint, meshes have been proposed as adjuncts to improve implant support and contour, although reported benefits and complication profiles vary across studies.^26^ In the present study, the absorbable synthetic mesh GORE^Ⓡ^ BIO-A^Ⓡ^ was used, which provides its main structural support during the early postoperative period, while undergoing progressive hydrolytic degradation over several months.^27^ Composed of polyglycolic acid (PGA) and trimethylene carbonate (TMC), this mesh was chosen mainly because it offers greater predictability in the healing process, lower risk of contamination, and reduced cost compared to biologic matrices.^28^ It is important to emphasize that the use of the synthetic mesh in this series reflected the institutional practice of the surgical team during the study period, rather than a general recommendation applicable to all implant-based breast procedures. Because this study was designed as a retrospective case series without a control group, it does not allow conclusions regarding the necessity, superiority, or comparative effectiveness of mesh use compared with standard techniques performed without mesh. The findings should therefore be interpreted within the context of this specific clinical experience.

Our study, to the best of our knowledge the first to use this matrix in breast reconstruction, was based on consolidated evidence of the application of GORE^Ⓡ^ BIO-A^Ⓡ^ in other surgical contexts that require structural support and effective stimulation of tissue repair. The mesh has been employed with positive outcomes in procedures such as hiatal and paraoesophageal hernia repair, abdominal wall reconstruction, and ventral or incisional hernia repair, settings in which its porous three-dimensional structure promotes cellular integration and predictable tissue remodeling.^29^^,^^30^

Among the main findings, it was observed that advanced age, overweight, multiple previous breast surgeries, and prior radiotherapy or chemotherapy were characteristics observed among patients whose breasts developed postoperative adverse events. Although these data are consistent with the literature, the low frequency of complications in this series (*n* = 5) warrants cautious interpretation. The association between age and postoperative complications has been reported in several studies, attributed to reduced tissue elasticity, slower healing, and a higher prevalence of comorbidities in older patients.^31^, ^32^, ^33^ In the present study, the mean age of patients who experienced complications was 55.2 years, compared with 45.8 years in the group without adverse outcomes, corroborating previous reports. With regard to BMI, although there is evidence indicating a higher incidence of infection, seroma, and dehiscence in overweight and obese patients, no significant difference in BMI was observed between the groups analyzed in this study. This suggests that other factors, such as adjuvant oncologic treatments, may have exerted a greater influence on the outcomes.

A key determinant of higher complication rates in reconstructive surgery, particularly in breast procedures, is the administration of radiotherapy and chemotherapy, mainly due to the induction of fibrotic changes, alterations in local vascularization, and impairment of tissue remodeling, which together result in suboptimal healing.^34^^,^^35^ In the present study, 80% of patients who developed complications had undergone radiotherapy and 100% had received adjuvant chemotherapy, findings that are consistent with previous reports.^36^^,^^37^ Another relevant observation was the higher incidence of complications among patients with a history of multiple breast surgeries. This factor may be related to reduced tissue reserve and alterations in breast histoarchitecture, compromising tissue integrity and increasing susceptibility to infection and seroma formation.^38^^,^^39^ In this study, most patients who experienced complications had undergone at least two prior breast procedures, reinforcing this hypothesis.

The temporal analysis of postoperative complications revealed that most events occurred between 30 and 90 days after surgery, a critical period for wound healing and tissue adaptation following breast reconstruction. These findings demonstrate a higher concentration of complications within the first postoperative month, but also highlight the possibility of late events during follow-up. It is noteworthy that all complications occurred in patients who underwent surgery for breast cancer, whereas no adverse events were observed among patients undergoing purely aesthetic procedures. However, this difference likely reflects the intrinsically lower baseline risk profile of aesthetic cases, which generally involve healthier tissues and the absence of prior oncologic treatments, rather than a specific protective effect of the mesh itself. Moreover, all complications were observed in the breast affected by the disease. Previous studies, such as those by Fischer et al.^40^ and Jagsi et al.,^41^ have reported that surgical complications frequently arise within the first 3 months, which is consistent with the present findings.

Despite presenting solid findings, this study has limitations that should be acknowledged. An important limitation of this study is the heterogeneity of the included population, which comprised post-mastectomy reconstructions, aesthetic mastopexies with implants, and contralateral symmetrization procedures. These surgical indications have substantially different baseline risk profiles, particularly regarding tissue quality, prior oncologic treatments, and surgical complexity. As a result, postoperative complication rates cannot be interpreted as directly comparable among these groups. Furthermore, the retrospective design and absence of a control group prevent any conclusions regarding the superiority, necessity, or risk reduction associated with the use of the synthetic mesh itself. Therefore, the findings should be interpreted as descriptive of initial clinical experience and indicative of feasibility and preliminary safety rather than comparative effectiveness. Its retrospective design may be subject to selection and information bias, as data were collected from medical records. In addition, the relatively small sample size may limit the generalizability of the results to broader populations. The absence of smokers in the cohort also prevented the evaluation of the impact of smoking on postoperative complications. Another limitation is that other clinical and surgical factors that could influence outcomes were not analyzed, such as the specific type of chemotherapy administered, the interval between oncologic treatments and breast reconstruction, and the presence of pre-existing comorbidities. Nevertheless, the study also has relevant strengths. It provides practical evidence of the performance of absorbable synthetic meshes in the context of breast reconstruction. the observation that most adverse events occurred in patients exposed to adjuvant chemotherapy underscores the importance of individualized surgical planning and close monitoring in oncologic patients. Furthermore, the combined analysis of variables such as age, BMI, previous treatments, and surgical indication enhances the understanding of factors that influence breast reconstruction outcomes and may help guide safer and more effective strategies.

## Conclusion

The use of synthetic absorbable mesh in breast reconstruction and aesthetic mastopexy was technically feasible and showed an acceptable preliminary safety profile within the limitations of this retrospective case series. Although a small number of postoperative complications occurred, these events appeared to be more closely related to patient and treatment factors than to the mesh itself. Given the retrospective design and absence of a control group, no conclusions can be drawn regarding comparative effectiveness or outcome improvement. Larger prospective studies with appropriate control groups and longer follow-up are needed to better define the role of this material in breast surgery.

## Ethical approval and informed consent

This study was designed as a retrospective case series; therefore, formal ethical approval and informed consent for participation were not required. However, all patients provided written consent for the use of their clinical photographs for publication purposes.

## Funding

This work received financial support from W. L. Gore & Associates do Brasil Ltda, which covered the publication fee. The funding source had no involvement in the study design, data collection, analysis, interpretation, or writing of the manuscript.

## Declaration of generative AI and AI-assisted technologies in the writing process

The authors did not use generative AI and AI-assisted technologies in the writing process.

## CRediT author statement

**LS:** Conceptualization, Methodology, Validation, Investigation, Resources, Writing – Original Draft. **BD:** Methodology, Investigation. **GC:** Methodology, Writing – Original Draft. **NS:** Methodology, Writing – Original Draft. **VS:** Writing – Review & Editing. **RL:** Writing – Review & Editing. **RR:** Writing – Review & Editing, Visualization. **GA:** Formal analysis, Data curation, Writing – Original Draft, Visualization. **DM:** Data curation, Writing – Review & Editing, Supervision, Project administration.

## Declaration of competing interest

The authors declare no conflict of interest.

## Appendix Supplementary materials

Image, application 1
