# Supplementary material for: Clinical outcomes of synthetic absorbable mesh use in breast surgery: First case series in reconstruction and aesthetic mastopexy
Source: JPRAS Open. 2026 Mar 27;49:566–78. doi: 10.1016/j.jpra.2026.03.030 (PMC13101545; doi:10.1016/j.jpra.2026.03.030)
Supplement: Supplementary file 1 [file mmc1.docx]

STROBE Statement—checklist of items that should be included in reports of observational studies

|  | Item No. | Recommendation | Page  No. | Relevant text from manuscript |
| --- | --- | --- | --- | --- |
| **Title and abstract** | 1 | (*a*) Indicate the study’s design with a commonly used term in the title or the abstract | p.1 (Title), p.2 (Abstract) | Title: “Clinical Outcomes… First Case Series…”; Abstract: “This cross-sectional study included women…” |
|  |  | (*b*) Provide in the abstract an informative and balanced summary of what was done and what was found | p.2 (Abstract) | Objectives, methods, results, conclusions all summarized with data (38 patients, 19.7% complications, mainly patient-related) |
| Introduction | | | |  |
| Background/rationale | 2 | Explain the scientific background and rationale for the investigation being reported | p.3–5 (Introduction) | Discusses burden of breast cancer, role of mastectomy, limitations of current reconstruction methods, rationale for synthetic mesh |
| Objectives | 3 | State specific objectives, including any prespecified hypotheses | p.6 (Introduction, last paragraph) | “…aimed to demonstrate feasibility of its use… analyze correlation with patient profiles… investigate potential complications…” |
| Methods | | | |  |
| Study design | 4 | Present key elements of study design early in the paper | p.7 (Methodology – Study Design) | Cross-sectional retrospective case series; period Oct 2022–Oct 2024; data from medical records |
| Setting | 5 | Describe the setting, locations, and relevant dates, including periods of recruitment, exposure, follow-up, and data collection | p.7 (Study Design) | Three hospitals in Americana, São Paulo, Brazil; Oct 2022–Oct 2024 |
| Participants | 6 | (*a*) *Cohort study*—Give the eligibility criteria, and the sources and methods of selection of participants. Describe methods of follow-up  *Case-control study*—Give the eligibility criteria, and the sources and methods of case ascertainment and control selection. Give the rationale for the choice of cases and controls  *Cross-sectional study*—Give the eligibility criteria, and the sources and methods of selection of participants | p.7–8 (Study Design/Participants) | Inclusion: women undergoing breast reconstruction or mastopexy with implants + mesh; data from records; informed consent waived |
|  |  | (*b*) *Cohort study*—For matched studies, give matching criteria and number of exposed and unexposed  *Case-control study*—For matched studies, give matching criteria and the number of controls per case | N/A | Not a matched design (cross-sectional). |
| Variables | 7 | Clearly define all outcomes, exposures, predictors, potential confounders, and effect modifiers. Give diagnostic criteria, if applicable | p.13 (Data Collection) | Outcomes: major vs minor complications; predictors: age, BMI, surgeries, radiotherapy, chemotherapy; all patients nonsmokers |
| Data sources/ measurement | 8* | For each variable of interest, give sources of data and details of methods of assessment (measurement). Describe comparability of assessment methods if there is more than one group | p.13 (Data Collection) | Medical records; BMI = height/weight; adjuvant treatments from records; complications classified as minor vs major |
| Bias | 9 | Describe any efforts to address potential sources of bias | p.7 (Methods – Study Design), p.27 (Discussion – Limitations) | Retrospective design acknowledged; data from records; limitations discussed |
| Study size | 10 | Explain how the study size was arrived at | p.7 (Study Design) | 38 patients (66 breasts); all consecutive cases within timeframe included |

Continued on next page

| Quantitative variables | 11 | Explain how quantitative variables were handled in the analyses. If applicable, describe which groupings were chosen and why | p.14 (Data Analysis) | p.14 (Data Analysis) |
| --- | --- | --- | --- | --- |
| Statistical methods | 12 | (*a*) Describe all statistical methods, including those used to control for confounding | p.14 (Data Analysis) | p.14 (Data Analysis) |
|  |  | (*b*) Describe any methods used to examine subgroups and interactions | p.14 (Data Analysis) | p.14 (Data Analysis) |
|  |  | (*c*) Explain how missing data were addressed | \| 16 (Results/Table 1) \| \| --- \| | \| 16 (Results/Table 1) \| \| --- \| |
|  |  | (*d*) *Cohort study*—If applicable, explain how loss to follow-up was addressed  *Case-control study*—If applicable, explain how matching of cases and controls was addressed  *Cross-sectional study*—If applicable, describe analytical methods taking account of sampling strategy | N/A | Not applicable, as all eligible cases included (no sampling). |
|  |  | (*e*) Describe any sensitivity analyses | N/A | None performed. |
| Results | | | | |
| Participants | 13* | (a) Report numbers of individuals at each stage of study—eg numbers potentially eligible, examined for eligibility, confirmed eligible, included in the study, completing follow-up, and analysed | p.15–16 (Results, Participants) | 38 patients, 66 breasts included; all eligible consecutive cases; no exclusions reported |
|  |  | (b) Give reasons for non-participation at each stage | N/A | Retrospective record review — not applicable. |
|  |  | (c) Consider use of a flow diagram | N/A | Not included (study size small, all cases included). |
| Descriptive data | 14* | (a) Give characteristics of study participants (eg demographic, clinical, social) and information on exposures and potential confounders | p.16–18 (Results, Table 1) | Demographic and clinical data: mean age 47, BMI, prior surgeries, adjuvant therapies |
|  |  | (b) Indicate number of participants with missing data for each variable of interest | p.16 (Table 1) | Missing BMI for 2 patients (5.3%). |
|  |  | (c) *Cohort study*—Summarise follow-up time (eg, average and total amount) | N/A | Cross-sectional study, no follow-up time. |
| Outcome data | 15* | *Cohort study*—Report numbers of outcome events or summary measures over time | p.19–21 (Results, Complications) | Overall complication rate 19.7% (7.6% major, 12.1% minor), detailed by type |
|  |  | *Case-control study—*Report numbers in each exposure category, or summary measures of exposure | - | - |
|  |  | *Cross-sectional study—*Report numbers of outcome events or summary measures | - | - |
| --Main results | 16 | (*a*) Give unadjusted estimates and, if applicable, confounder-adjusted estimates and their precision (eg, 95% confidence interval). Make clear which confounders were adjusted for and why they were included | p.21–23 (Results, Logistic Regression) | Unadjusted and adjusted odds ratios reported for age, prior surgeries, radiotherapy, chemotherapy |
|  |  | (*b*) Report category boundaries when continuous variables were categorized | p.16–18 (Table 1) | BMI categories defined; age presented as continuous and categorical. |
|  |  | (*c*) If relevant, consider translating estimates of relative risk into absolute risk for a meaningful time period | N/A | Not applied (cross-sectional design, descriptive risk only). |

Continued on next page

| Other analyses | 17 | Report other analyses done—eg analyses of subgroups and interactions, and sensitivity analyses | p.23 (Results, Subgroup Analyses) | Complications stratified by oncologic vs aesthetic indication; by prior treatments |
| --- | --- | --- | --- | --- |
| Discussion | | | | |
| Key results | 18 | Summarise key results with reference to study objectives | p.25 (Discussion – first paragraph) | Main finding: mesh is safe, low complications, mostly related to patient comorbidities |
| Limitations | 19 | Discuss limitations of the study, taking into account sources of potential bias or imprecision. Discuss both direction and magnitude of any potential bias | p.27 (Discussion – Limitations) | Retrospective design, small sample size, absence of smokers, reliance on records |
| Interpretation | 20 | Give a cautious overall interpretation of results considering objectives, limitations, multiplicity of analyses, results from similar studies, and other relevant evidence | p.26–28 (Discussion) | Results interpreted cautiously; compared with literature; clinical relevance highlighted |
| Generalisability | 21 | Discuss the generalisability (external validity) of the study results | p.28 (Discussion – External validity) | Limited generalisability due to retrospective design and small, single-region cohort |
| Other information | |  | | |
| Funding | 22 | Give the source of funding and the role of the funders for the present study and, if applicable, for the original study on which the present article is based | \|  \| \| --- \|  \| p.30 (Funding statement) \| \| --- \| | Supported by W. L. Gore & Associates do Brasil Ltda (publication fee only, no role in study design or analysis) |

*Give information separately for cases and controls in case-control studies and, if applicable, for exposed and unexposed groups in cohort and cross-sectional studies.

**Note:** An Explanation and Elaboration article discusses each checklist item and gives methodological background and published examples of transparent reporting. The STROBE checklist is best used in conjunction with this article (freely available on the Web sites of PLoS Medicine at http://www.plosmedicine.org/, Annals of Internal Medicine at http://www.annals.org/, and Epidemiology at http://www.epidem.com/). Information on the STROBE Initiative is available at www.strobe-statement.org.
